# Global analysis of protein lysine succinylation profiles in common wheat

**DOI:** 10.1186/s12864-017-3698-2

**Published:** 2017-04-20

**Authors:** Yumei Zhang, Guangyuan Wang, Limin Song, Ping Mu, Shu Wang, Wenxing Liang, Qi Lin

**Affiliations:** 1 College of Agronomy and Plant Protection, Qingdao Agricultural University, Qingdao, Shandong 266109 China; 2 College of Life Sciences, Shandong Province Key Laboratory of Applied Mycology, Qingdao Agricultural University, Qingdao, 266109 China; 3 College of Agronomy, Shenyang Agricultural University, Shenyang, Liaoning 110866 China

**Keywords:** Lysine succinylation, Succinylome, *Triticum aestivum* L., Post-translational modification, Photosynthesis

## Abstract

**Background:**

Protein lysine succinylation is an important post-translational modification and plays a critical regulatory role in almost every aspects of cell metabolism in both eukaryotes and prokaryotes. Common wheat is one of the major global cereal crops. However, to date, little is known about the functions of lysine succinylation in this plant. Here, we performed a global analysis of lysine succinylation in wheat and examined its overlap with lysine acetylation.

**Results:**

In total, 330 lysine succinylated modification sites were identified in 173 proteins. Bioinformatics analysis showed that the modified proteins are distributed in multiple subcellular compartments and are involved in a wide variety of biological processes such as photosynthesis and the Calvin-Benson cycle, suggesting an important role for lysine succinylation in these processes. Five putative succinylation motifs were identified. A protein interaction network analysis revealed that diverse interactions are modulated by protein succinylation. Moreover, 21 succinyl-lysine sites were found to be acetylated at the same position, and 33 proteins were modified by both acetylation and succinylation, suggesting an extensive overlap between succinylation and acetylation in common wheat. Comparative analysis indicated that lysine succinylation is conserved between common wheat and *Brachypodium distachyon*.

**Conclusions:**

These results suggest that lysine succinylation is involved in diverse biological processes, especially in photosynthesis and carbon fixation. This systematic analysis represents the first global analysis of lysine succinylation in common wheat and provides an important resource for exploring the physiological role of lysine succinylation in this cereal crop and likely in all plants.

**Electronic supplementary material:**

The online version of this article (doi:10.1186/s12864-017-3698-2) contains supplementary material, which is available to authorized users.

## Background

Post-translational modifications (PTMs) play an important role in modulating diverse cellular processes, which are dynamic and reversible modifications of proteins during or after protein biosynthesis [1]. PTMs can change protein functions by introducing new functional groups such as acetyl, phospho, ubiquityl, succinyl and methyl groups. Among these changes, acetylation of lysine has been extensively studied in both eukaryotes and prokaryotes [2, 3]. Similar to lysine acetylation, lysine succinylation is one of the newly discovered PTMs that has been found in both eukaryotes and prokaryotes [1]. As one important PTM of proteins, lysine succinylation was defined as the transfer of a succinyl group to a lysine residue of a protein molecule [4].

Lysine succinylation was firstly discovered in histone proteins, and its role has been investigated in the regulation of gene transcription [5]. In addition to histones, other proteins in the nucleus, cytoplasm and mitochondria were also found to be succinylated [1, 6–8], indicating that lysine succinylation potentially regulates a wide variety of important biological processes. Advancements in liquid chromatography-mass spectrometry (LC-MS/MS) and high affinity purification of succinylated peptides have made it possible to study lysine succinylation on a proteomic scale. As a result, a large number of lysine-succinylated proteins have been identified in both prokaryotes [1, 6, 7, 9, 10] and eukaryotes [8, 9, 11–14]. These proteome-wide analyses of lysine succinylation revealed the broad role of this modification in various cellular pathways.

Common wheat (*Triticum aestivum* L.), which is also known as bread wheat, is one of the most important cereal crops in the world. Our previous proteomics analysis indicates that lysine acetylation is involved in regulating diverse biological processes in common wheat [15]. As one modification that happens on lysine residue and competes with acetylation, lysine succinylation of proteins is expected to play a critical role in wheat development and metabolism. To test this hypothesis, we performed the first proteomics study on lysine succinylation in common wheat. A total of 330 lysine succinylation sites in 173 proteins were identified. The modified proteins were localized in multiple compartments including the cytosol, chloroplast, mitochondria, nucleus, peroxisome, cytoskeleton, endoplasmic reticulum and extracellular and plasma membranes, suggesting that lysine succinylation can regulate various biological processes. Importantly, many proteins involved in photosynthesis and the Calvin-Benson cycle were found to be succinylated. We further compared the lysine succinylation data with the acetylome of common wheat for the modification sites and proteins, and 33 proteins were found to be modified by both acetylation and succinylation. Seven enzymes of these 33 proteins were involved in the Calvin-Benson cycle, suggesting that both types of modification may play important roles in regulating the carbon fixation metabolism process, especially the Calvin-Benson cycle. Comparative analysis of lysine succinylation profiles between common wheat and *Brachypodium distachyon* suggest that lysine succinylation plays both common and specific roles in different plant species. These findings provide a rich dataset for further functional analysis of lysine succinylation in this important cereal crop.

## Methods

### Plant material and growth conditions

The seedlings of common wheat variety (*T. aestivum* L.) Qing Mai 6 were grown in a greenhouse with the temperature set at 22/18 °C (day/night) and a photoperiod of 16/8 h (light/dark) [16]. The leaves were excised from 3-week-old seedlings and immediately used for protein extraction.

### Protein extraction and trypsin digestion

Proteins were extracted from common wheat leaves as previously described [15, 17]. Briefly, leaves excised from seedlings were ground in liquid nitrogen and sonicated three times in lysis buffer (8 M urea, 1% Triton-100, 10 mM dithiothreitol (DTT) and 1% Protease inhibitor cocktail) [17]. The remaining debris was removed by centrifugation at 20,000 × g for 15 min at 4 °C and proteins in the supernatant were precipitated with cold 15% trichloroacetic acid for 2 h at 4 °C. Proteins obtained above were redissolved in buffer (8 M urea, 100 mM (NH_4_)_2_CO_3_, pH 8.0). The protein solution was alkylated with 20 mM iodoacetamide for 45 min at room temperature in darkness following reducing with 10 mM DTT for 1 h at 37 °C [15, 17]. After dilution with (NH_4_)_2_CO_3_ to reduce urea concentration to less than 2 M, a two-step trypsin digestion was carried out with the method of Zhou et al. [15, 17].

### HPLC fractionation and affinity enrichment

After trypsin digestion, 10 mg of peptides were fractionated into 80 fractions by high pH reverse-phase HPLC using Agilent 300Extend C18 column and the separated peptides were then combined into 6 fractions [17]. For affinity enrichment, the fractions of peptide were incubated with pan anti-succinyllysine antibody conjugated agarose beads (PTM Biolabs) in NETN buffer (100 mM NaCl, 1 mM EDTA, 50 mM Tris-HCl, 0.5% NP-40, pH 8.0) for 12 h [17]. After washing four times with NETN buffer and twice with double distilled water, the lysine succinylation peptides bound to the agarose beads were eluted with 0.1% trifluoroacetic acid [15, 17].

### LC-MS/MS analysis

After cleaning with C18 ZipTips (Millipore), the enriched succinylated peptides were analyzed using mass spectrometer (Thermo Scientific^TM^ Q Exactive^TM^ Plus) as described [12, 17]. In brief, the peptides were firstly dissolved in 0.1% formic acid (FA) and separated using a reversed-phase analytical column (Acclaim PepMap RSLC, Thermo Scientific) on an EASY-nLC 1000 UPLC system [12]. Then, the peptides were analyzed by tandem mass spectrometry (MS/MS) in Q Exactive^TM^ Plus (Thermo Scientific) coupled online to the UPLC system. Detection of intact peptides were performed in the Orbitrap at a resolution of 70,000 (m/z 200) with NCE setting of 30. To scan MS, the m/z range was set from 350 to 1800 [12, 17]. The voltage for electrospray analysis was set at 2.0 kV [12, 18].

### Data analysis

The obtained MS/MS data of succinylation peptides was processed using MaxQuant with integrated Andromeda search engine (v.1.4.1.2) [19, 20]. The corresponding tandem mass spectra were searched against *UniProt_Triticum* database (146,090 sequences, released March, 2015) concatenated with reverse decoy database [17]. The parameters in MaxQuant were listed below: (1) variable modification: succinylation (lysine), acetylation (protein N-terminus), Oxidation (methionine); (2) fixed modification: carbamidomethylation (Cysteine); (3) digestion mode: trypisin/P; (4) maximal missed cleavages: 2; (5) first search PPM: 20; (6) main search PPM: 5; (7) maximal number of modifications per peptide: 5; (8) minimal peptide length: 7; (9) minimal score for modified peptides: 40; (10) maximal charges: 5 [17]. The thresholds of false discovery rate (FDR) were set at 1% for modification site, peptide and protein [17]. Firstly the MaxQuant generates a list of all peptides resulting from tryptic digestion of the common wheat proteins taking into account of all possible combinations of preset modifications, including succinylation. The MaxQuant then tries to match and score the MS/MS spectrum to these candidate peptides sequences. After some statistical processes including score and FDR limitation, the identified peptides sequences could be obtained from the raw MS/MS data. Then, the peptide search engineer will map these identified peptides to their corresponding proteins [19, 20].

### Bioinformatics analyses

The proteome of GO annotation was derived from the UniProt-GOA database (http://www.ebi.ac.uk/GOA/) [15]. Classification of the succinylated proteins in common wheat by GO annotation was carried out on the basis of biological process, cellular component and molecular function [14]. Functional description of identified protein domains and protein pathways were annotated by InterProScan and Kyoto Encyclopedia of Genes and Genomes (KEGG) database, respectively [17]. The GO, pathway and domain with a corrected *p*-value < 0.05 were considered significant [15]. WOLFPSORT was used to predict subcellular localization of the succinylated proteins in common wheat [14]. Motif analysis of lysine succinylation sites was analyzed by the software Motif-x and a position-specific heat map was generated by using the “heatmap.2” function from the “gplots” R-package [12–14]. NetSurfP was used to predict secondary structures of the succinylated wheat proteins [15, 21]. Protein-protein interaction (PPI) for the identified succinylated proteins were performed using Cytoscape software and a PPI network was generated from the STRING database according to the methods described previously [22, 23]. BLASTP was conducted to evaluate the conservation of lysine succinylated proteins between common wheat and *Brachypodium distachyon* [13].

### Immunoprecipitation and Western blot analysis

Total proteins were extracted from the leaves of common wheat as previously described [24]. One mg of soluble protein was incubated in the presence or absence of 2 μg of catalase 1 antibody (Agrisera) at 4 °C for 2 h. Protein A agarose beads were subsequently added and the mixture was incubated for 1 h. The beads were washed 5 times with 500 μl of lysis buffer, and the bound proteins were eluted with 1× SDS-PAGE loading buffer (50 mM Tris-HCl, pH 6.8, 2% SDS, 10% glycerol, 2% 2-mercaptoethanol, 0.01% bromophenol blue) [24].

Proteins were resolved on a 10% gel and subjected to immunoblotting [25]. Catalase 1 and acetylated catalase 1 were detected by catalase 1 antibody (1:10,000 dilution) and anti-succinyl lysine antibody (1:1000 dilution, PTM Biolabs), respectively. Proteins were visualized using Immobilon Western Chemiluminescent HRP Substrate (Millipore) according to the manufacturer’s protocol [13, 14].

## Results and discussion

### Proteome-wide analysis of lysine succinylation sites and proteins in common wheat

Lysine succinylation is a newly discovered PTM that occurs in both prokaryotes and eukaryotes [9], but its regulatory role is poorly studied in common wheat, one of the most important crops in the world. To map the lysine succinylation sites in wheat, a proteome-wide method was used. Proteins were extracted and digested into peptides by trypsin as described in “Methods”. The succinylation peptides were then immune affinity-purified and analyzed using high-resolution LC-MS/MS. An overview of the experimental procedures used in this study was shown in Additional file 1: Figure S1a. To confirm the reliability of the MS data, the mass error of all the identified peptides were checked. The distribution of mass error was near zero and most values were less than 5 PPM, which means that the mass accuracy of the MS data fits the requirement (Additional file 1: Figure S1b). In addition, the length of most peptides was between 8 and 32, which is consistent with the properties of tryptic peptides (Additional file 1: Figure S1c). Therefore, sample preparation meets the standard. Using this method, a total of 330 lysine succinylation sites in 173 protein groups were identified (Additional file 2: Table S1). Note that 5542 peptides were obtained and 329 of them were found to be succinylated (Additional file 2: Table S1). Catalase 1, one of the most important enzymes responsible for removing reactive oxygen species, was identified to be succinylated on the lysine residue, K481 (Additional file 1: Figure S2a). Immunoprecipitation and Western blot analysis validated succinylation of catalase 1 in the leaves of common wheat (Additional file 1: Figure S2b). To assess the distribution of succinylation sites in the identified wheat proteins, we calculated the numbers of modification sites per protein. The results in Additional file 1: Figure S1d showed that for these identified proteins, 64.2% (111) of them have only one succinylated lysine site, whereas 35.8% (62) was modified on multiple lysine residues. These results, together with the identification of succinylated proteins in tomato (*Solanum lycopersicum*), stiff brome (*Brachypodium distachyon* L.) and rice [12–14], suggest that lysine succinylation is a widespread phenomenon is plants.

### Motif analysis of lysine succinylation sites

**Fig. 1:**
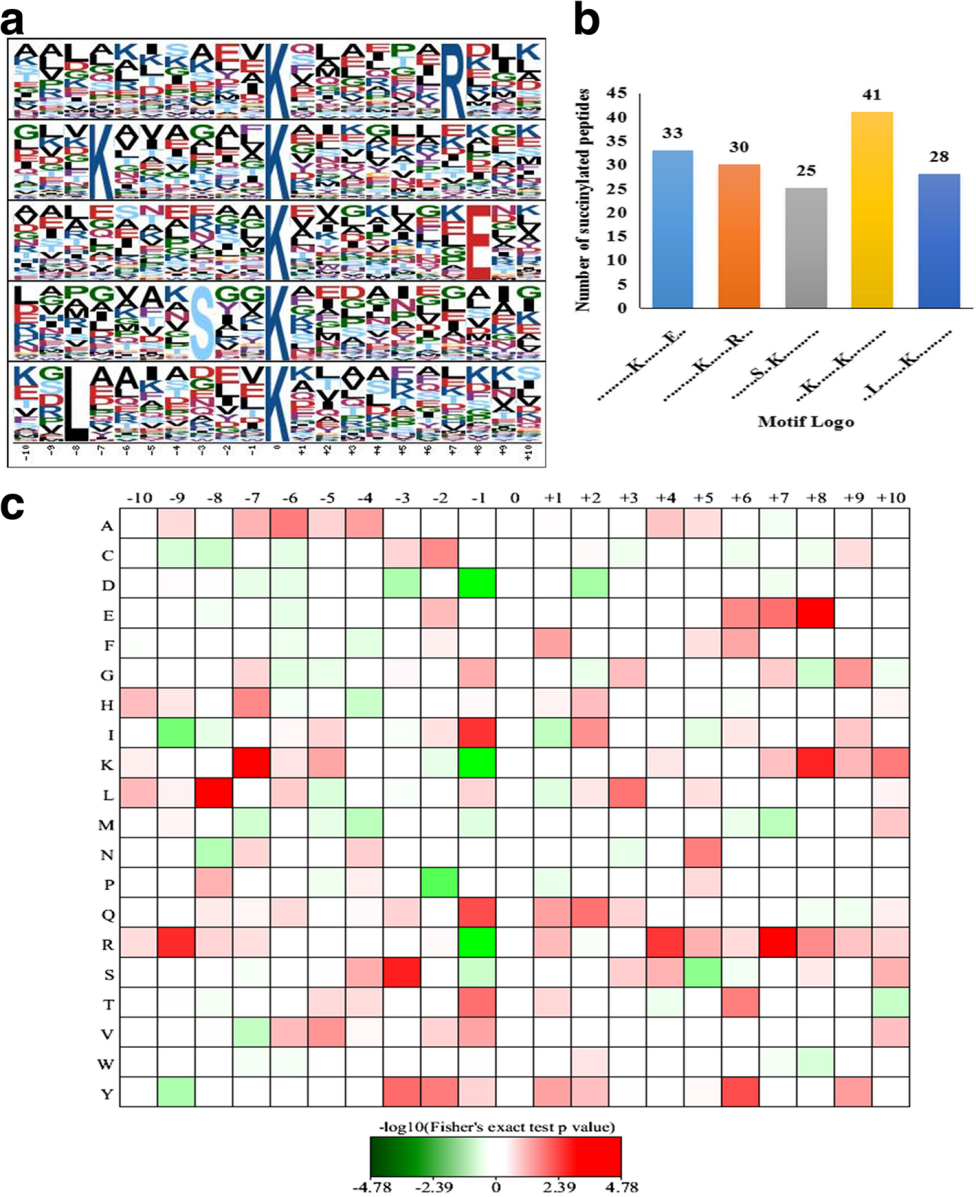
Properties of lysine succinylated peptides. **a** Succinylation sequence motifs for ±10 amino acids around the lysine succinylation sites. **b** Number of peptides containing each of the conserved motifs. **c** Heat map of the amino acid compositions of the succinylated sites

To evaluate the nature of lysine succinylation sites in wheat, we investigated the sequence motifs in the identified peptides with the Motif-x program. Five conserved sequences, with amino acid sequences from −10 to +10 surrounding the succinylated lysine, were extracted from 157 succinylated peptides (Fig. 1). These motifs were K^su^******R, K******K^su^, K^su^*******E, S**K^su^ and L******K^su^ (K^su^ indicates the lysine succinylation site and * represents an unspecified amino acid residue) (Fig. 1a), and they exhibited different abundances (Fig. 1b). In accordance with these findings, the results of heat map showed that the frequencies of leucine (L) in position −8, lysine (K) in position −7, serine (S) in position −3, arginine (R) in position +7 and glutamic acid (E) in position +8 were highest, whereas the occurrence of aspartic acid (D), K and R was lowest in position −1 (Fig. 1c). Therefore, proteins with particular amino acid residues in the corresponding positions are more likely to be modified with succinyl groups in wheat. It is noteworthy that two of these succinylation motifs, K^su^******R and K******K^su^, were also observed in tomato [12] and the marine bacterium *V. parahemolyticus* [1], confirming that lysine succinylation is a highly conserved post-translational modification among different species.

### Secondary structure analysis of acetylated proteins

To elucidate the relationship between lysine succinylation and protein structure in wheat, a structural analysis of all the identified proteins was performed. The results in Additional file 1: Figure S3a showed that 34.3% (113) of the lysine succinylation sites were located in regions with ordered secondary structures. Among them, 27.8% (92) were located in alpha-helix and 6.5% (21) were in beta-strand (Additional file 1: Figure S3a). In addition, 65.7% (217) of the lysine succinylation sites were distributed in regions predicted to be disordered of proteins (Additional file 1: Figure S3a). However, given that the distribution pattern of succinylated lysines and all lysines is similar (Additional file 1: Figure S3a), there seem to be no tendency of succinylation in common wheat. We further evaluated the surface accessibility of lysine succinylation sites. The results showed that, in comparison with 40.46% of all lysine residues, 38.07% of lysine succinylation sites were exposed to protein surface (*p* = 0.0636) (Additional file 1: Figure S3b). Therefore, the surface property of proteins is unlikely to be affected by lysine succinylation.

### Functional annotation and cellular localization of succinylated proteins in common wheat

**Fig. 2:**
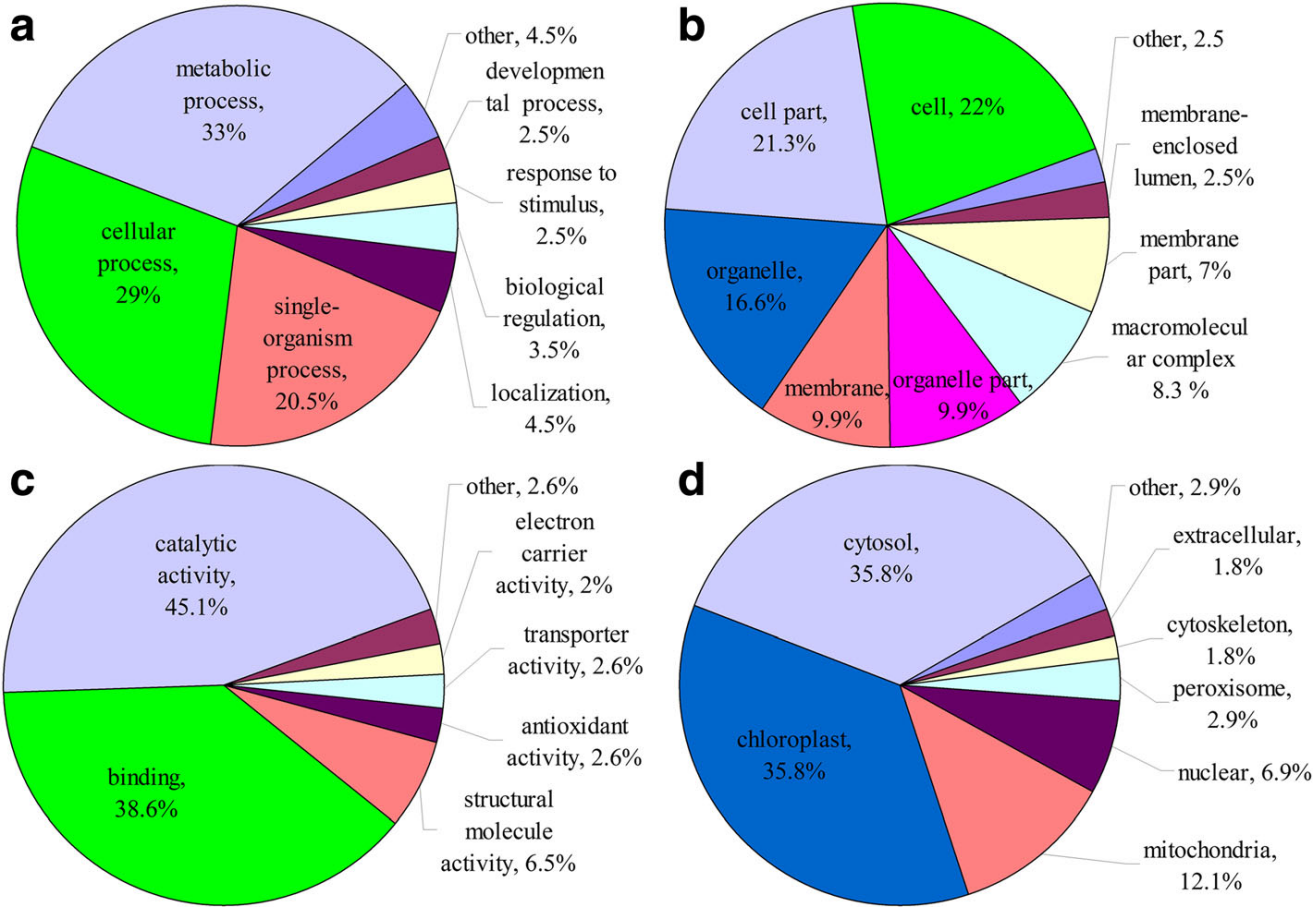
Pie charts showing the functional classification of succinylated proteins in common wheat. **a** Classification of the succinylated proteins based on biological process. **b** Classification of the succinylated proteins based on cellular component. **c** Classification of the succinylated proteins based on molecular function. **d** Subcellular localization of the succinylated proteins

To better elucidate the potential roles of lysine succinylation in wheat, we submitted all the succinylated proteins to a Gene Ontology (GO) functional classification analysis on the basis of their biological process, cellular component and molecular function (Fig. 2a–c). The biological process analysis indicated that most of the succinylated proteins were involved in metabolic processes (33%) and cellular processes (29%) (Fig. 2a). The results of cellular component analysis showed that a large number of the modified proteins were distributed within the cell (22%), cell part (21.3%), organelles (16.6%), membrane (9.9%) and organic part (9.9%) (Fig. 2b). This distribution pattern is not surprising at all because a large proportion of succinylated proteins identified in other organisms are involved in metabolic processes [12–14]. Consistent with these findings, most succinylated proteins were found to be associated with catalytic and binding activities in the molecular function classification, accounting for 45.1 and 38.6% of all the identified proteins, respectively (Fig. 2c).

Subcellular localization of the succinylated proteins was also investigated. As shown in Fig. 2d, most of the succinylated proteins in common wheat were distributed in the cytosol (35.8%), chloroplast (35.8%), mitochondria (12.1%) and nucleus (6.9%). These results, together with the data on GO functional classification, suggest that the succinylated proteins, located to diversified cellular compartments, are involved in numerous biological processes in wheat.

### Functional enrichment analysis of succinylated proteins

**Fig. 3:**
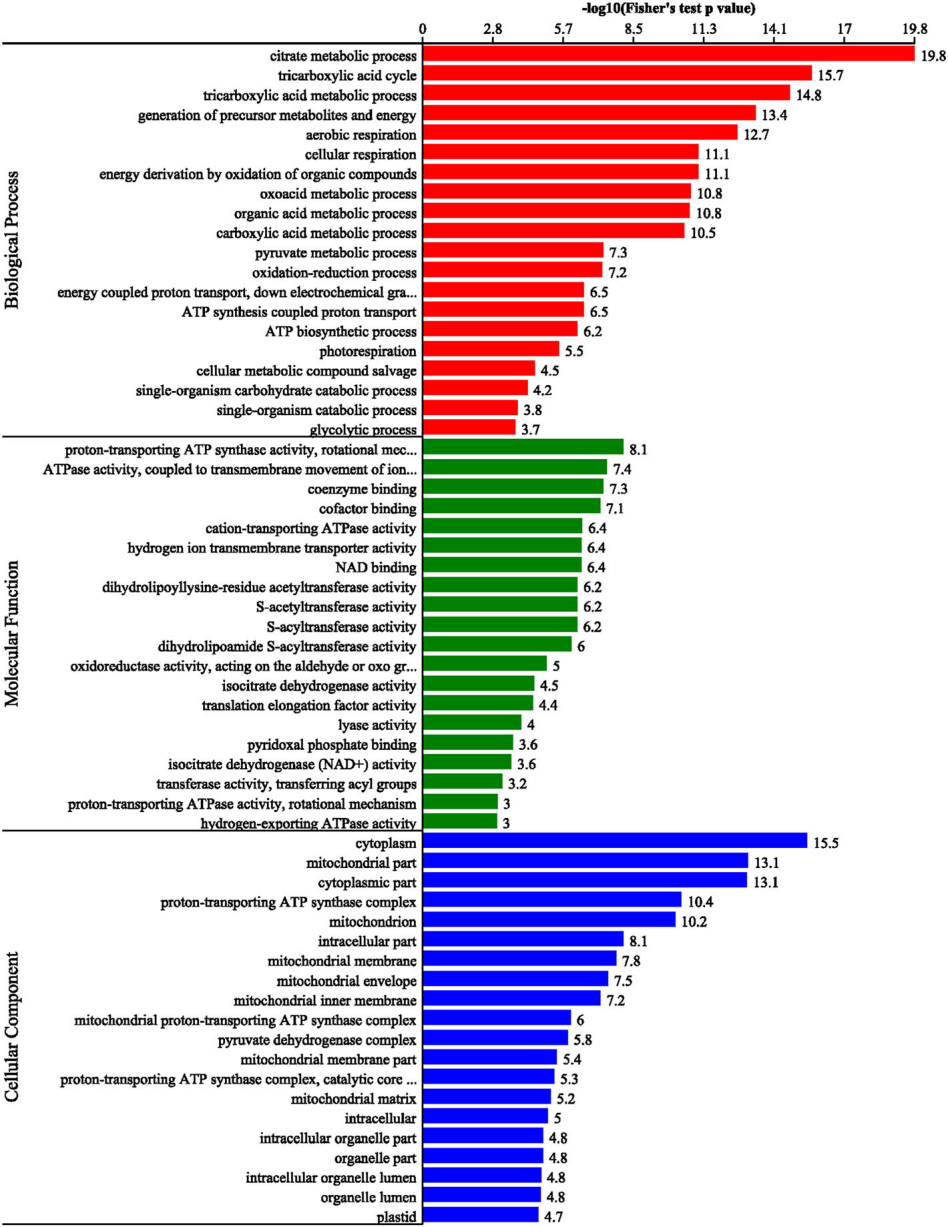
GO-based enrichment analysis in terms of biological process (*red bars*), molecular function (*green bars*) and cell component (*blue bars*)

To further understand the characteristics of succinylated proteins in common wheat, functional enrichment of GO (biological process, molecular function and cellular component), KEGG pathway and protein domain analyses were performed (Fig. 3, Additional file 2: Table S2–S4). The results of biological process enrichment showed that most of the succinylated proteins were involved in metabolic processes and energy generation (Fig. 3, red bars, Additional file 2: Table S2). In agreement with these observations, many modified proteins were found to be associated with enzymatic and binding activities in molecular function enrichment analysis (Fig. 3, green bars, Additional file 2: Table S3). Consistent with these findings, proteins located to the cytoplasm, mitochondrial and proton-transporting ATP synthase complex were more likely to be succinylated based on cellular component enrichment analysis (Fig. 3, blue bars, Additional file 2: Table S4).

Similar results were also obtained in the domain and KEGG pathway enrichment analyses. The results of domain enrichment analysis showed that proteins with domains of 2-oxo acid dehydrogenase, biotin/lipoyl attachment, mitochondrial carrier, NAD(P)-binding and ATPase were more prone to succinylation (Additional file 1: Figure S4a, Additional file 2: Table S5). In support of these findings, KEGG pathway enrichment analysis indicated that most of the succinylated proteins were related to TCA cycle, carbon fixation and glycolysis/gluconeogenesis (Additional file 1: Figure S4b, Additional file 2: Table S6).

Taken together, wide distribution of lysine succinylated proteins invovled in diverse pathways suggests an important role of this post-translational modification in cell metabolism.

### Analysis of succinylated proteins involved in photosynthesis and carbon fixation

**Fig. 4:**
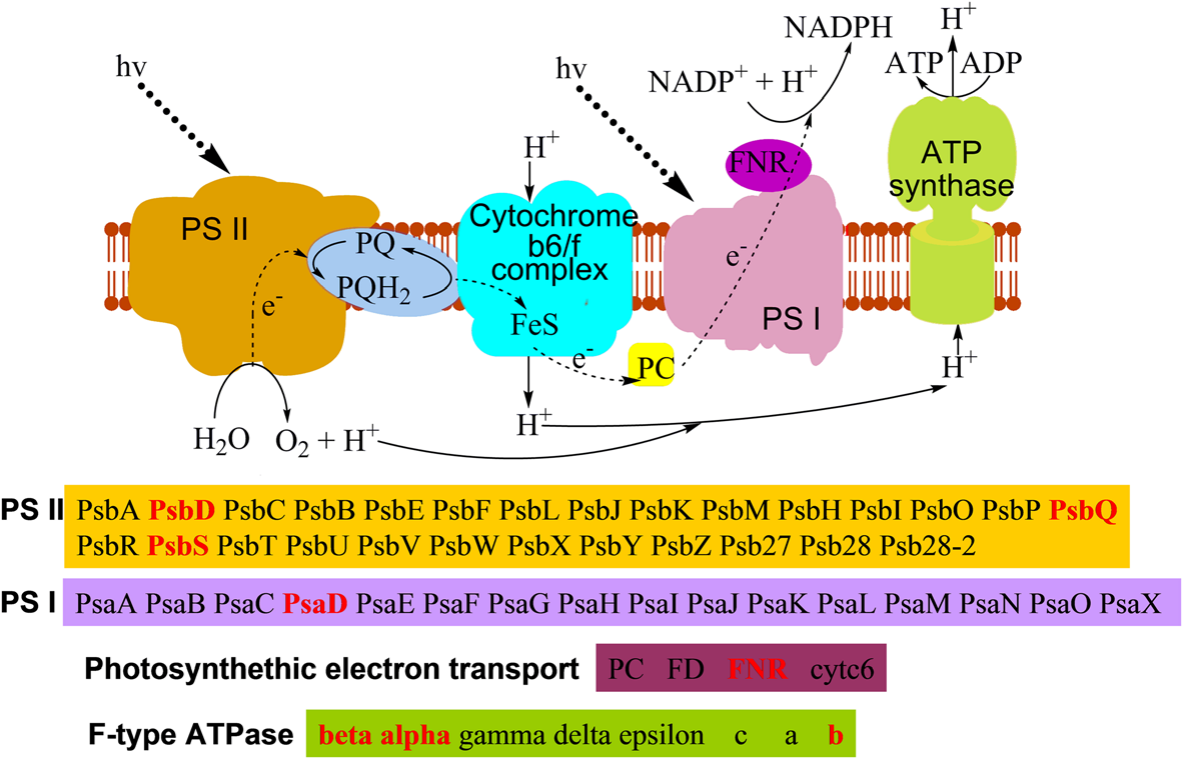
Succinylated proteins involved in photosynthesis. The identified succinylated proteins were highlighted in red

In wheat, photosynthesis is one of the most important metabolic processes. Photosynthesis, taking place in the chloroplast, converts light energy to chemical energy and stores the latter in the bonds of sugar [15]. The fact that 35.8% of the succinylated proteins in common wheat are located to the chloroplast (Fig. 2d), together with the enrichment of succinylated proteins in energy metabolic processes (Fig. 3), suggest that lysine succinylation may play an important role in photosynthesis. In agreement with this hypothesis, eight proteins involved in photosynthesis were found to be succinylated, including one subunit of Photosystem I (PsaD), three subunits of Photosystem II (PsbD, PsbQ and PsbS) and three different subunit types of ATP synthase (alpha, beta and epsilon-b) (Fig. 4). In addition, lysine succinylation also occurs in FNR (Ferredoxin-NADP^+^ oxidoreductase) (Fig. 4), which catalyzes the reversible electron transfer between Fd and NAD(P)H [26]. Consistent with our results, several proteins involved in photosynthesis were also found to be lysine succinylated in leaves of *B. distachyon* L. [13].

The products of photosynthesis, such as ATP and NADPH, then participate in carbon fixation. As such, we further investigated the succinylated proteins involved in carbon fixation in common wheat. Not unexpectively, eleven enzymes involved in carbon fixation were found to be succinylated (Additional file 1: Figure S6). Among these enzymes, ten are associated with the Calvin-Benson cycle (Additional file 1: Figure S6). Moreover, two enzymes involved in the C4-dicarboxylic acid cycle, namely malate dehydrogenase [EC:1.1.1.37] and alanine transaminase [EC:2.6.1.2], were also among the succinylated proteins in common wheat (Additional file 1: Figure S6). Collectively, these findings support the notion that lysine succinylation could be an important part of the regulatory mechanism underlying the processes of photosynthesis and the carbon metabolism in common wheat.

### PPI network of succinylated proteins in wheat

**Fig. 5:**
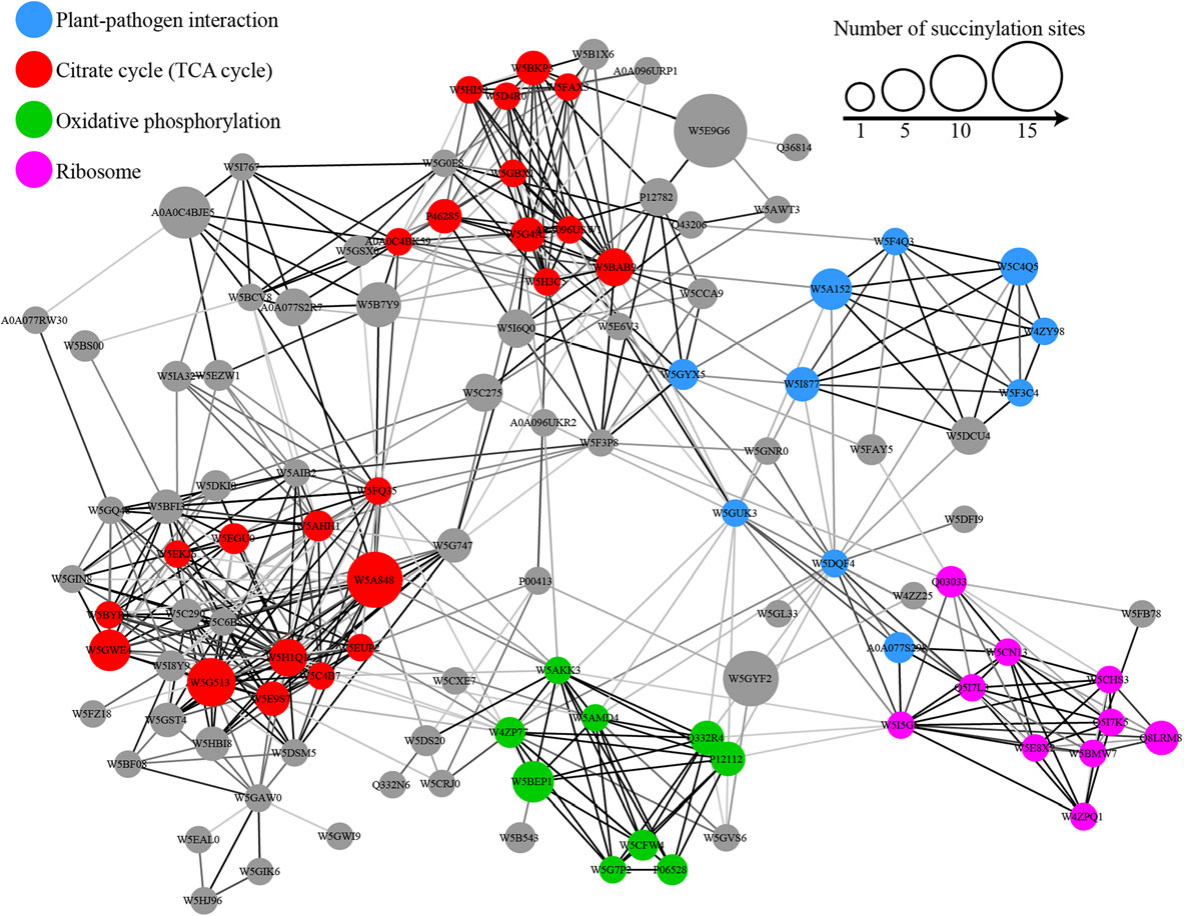
Interaction networks of succinylated proteins in common wheat

To investigate the cellular processes regulated by succinylation in common wheat, we established a PPI network. As shown in Fig. 5, a total of 116 succinylated proteins were mapped to the PPI network (Additional file 2: Table S7–S8), which demonstrates how these proteins modulate diversified pathways in common wheat. Six highly interconnected clusters of succinylated proteins were retrieved according to the algorithm in Cytoscape software (Additional file 2: Table S7). The top four clusters (Cluster I-IV) identified were the citrate cycle (Cluster I), ribosome (Cluster II), oxidative phosphorylation (Cluster III) and plant-pathogen interaction (Cluster IV) (Fig. 5, Additional file 1: Figure S5a–d). The complicated interaction networks of succinylated proteins indicate that the physiological interactions among these protein complexes are likely to contribute to their coordination and cooperation in this important cereal crop.

### Overlap between lysine succinylation and acetylation in common wheat

**Fig. 6:**
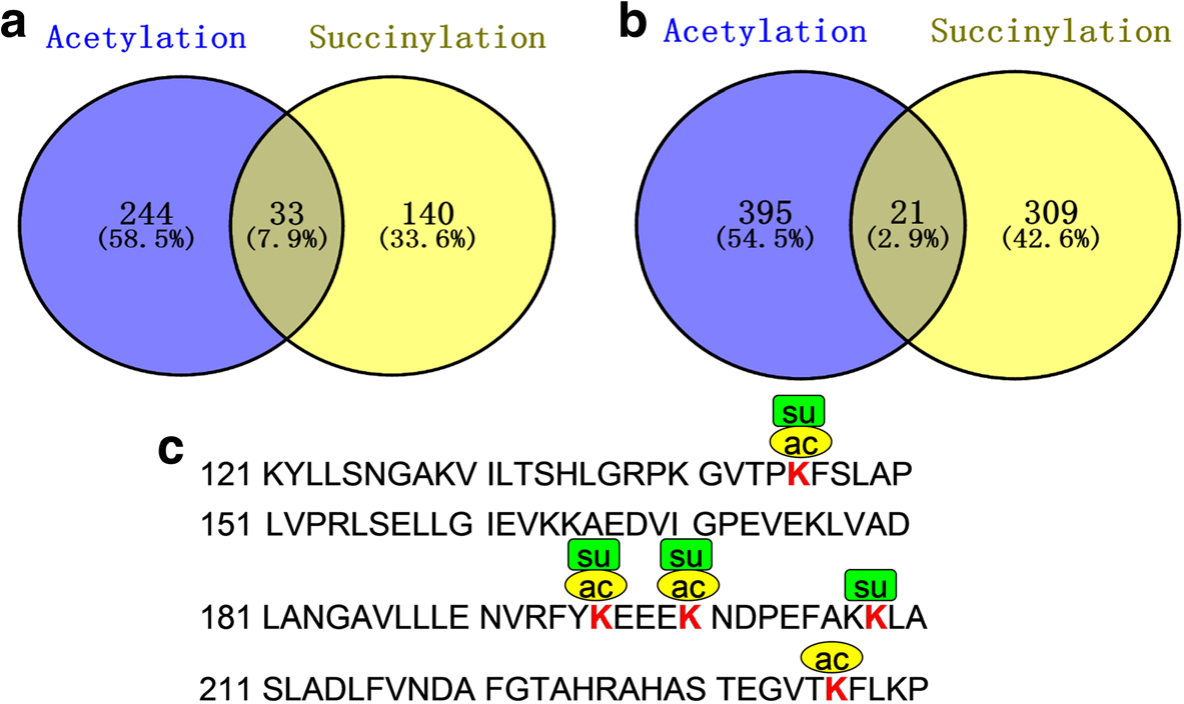
Overlap between lysine succinylation and lysine acetylation in wheat. **a** Overlap of succinylated proteins and acetylated proteins. **b** Overlap of succinylated sites and acetylated sites. **c** A representative protein showing the overlap of acetylation and succinylation sites. The three lysine sites at 145, 196, and 200 are both modified by acetylation (ac) and succinylation (su) in phosphoglycerate kinase (Entry ID: P12782)

As one type of PTMs, lysine succinylation competes with other modifications, such as acetylation, for lysine residues [6]. In our previous acetylome research, 416 acetylated lysine sites were identified on 277 proteins in wheat [15]. To determine whether acetylation and succinylation can occur on the same lysine residue, we compared the lysine succinylome with the acetylome obtained previously in our laboratory [15]. As shown in Fig. 6a and Additional file 2: Table S9, 33 proteins were modified by both acetylation and succinylation, which account for 7.9% of the total modified proteins (417). We also found that 21 succinylation sites were acetylated at the same position, accounting for 2.9% of the total identified sites (Fig. 6b, Additional file 2: Table S10). A representative sample of overlapping between acetylation and succinylation was shown in Fig. 6c. In the enzyme known as phosphoglycerate kinase, 4 succinylation sites at K145, K196, K200 and K208 were identified, and three sites at K145, K196 and K200 were also determined to be acetylated (Fig. 6c). Moreover, among the 10 succinylated enzymes involved in the Calvin-Benson cycle, 7 of them were found to be acetylated (Additional file 1: Figure S6). These findings suggest that succinylation and acetylation can occur on the same lysine residue and may coordinately regulate the function of many proteins in common wheat.

### Comparative analysis of lysine succinylation profiles between common wheat and *Brachypodium distachyon*

**Fig. 7:**
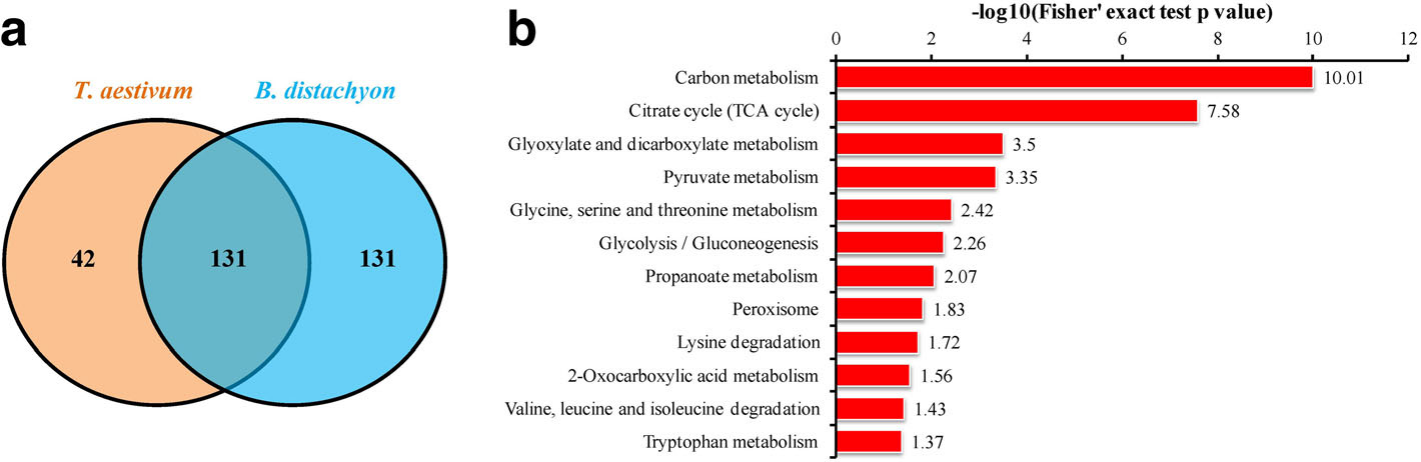
Comparative analysis of lysine succinylation profiles between common wheat and *B. distachyon*. a Venn diagram showing common succinylated proteins identified in wheat and *B. distachyon*. b KEGG pathway-based enrichment analysis of common succinylated proteins identified in wheat and *B. distachyon*

Common wheat and *Brachypodium distachyon* are both members of the Pooideae subfamily. To reveal the commonality and specificity of lysine succinylation in these grasses, we used the sequences of the identified proteins in common wheat to perform a BLAST search and estimated the degree of conservation of succinylated proteins between these plants. The parameters were set as follows: E-value < 1 × 10^−10^, score ≥ 80, and identity ≥ 60%. As shown in Fig. 7a, 131 succinylated proteins identified in common wheat (75.7%) had homologous proteins in *B. distachyon* (50.0%), with an average identity of 92% (Additional file 2: Table S11). Most of the homologous proteins are involved in carbon metabolism, TCA cycle, Glycolysis/Gluconeogenesis and protein/amino acids metabolism (Fig. 7b, Additional file 2: Table S12), indicating the crucial role and conservation of lysine succinylation in both plants. Although a number of proteins in the pathways of carbon fixation, photosynthesis and oxidative phosphorylation were found to be succinylated in both common wheat (Additional file 1: Figure S4b, Additional file 2: Table S6) and *B. distachyon* [13], few common proteins were identified between them. Moreover, succinylated proteins associated with peroxisome were only identified in common wheat (Additional file 1: Figure S4b, Additional file 2: Table S6), and modified proteins related to microbial metabolism in diverse environments and biosynthesis of antibiotics were only found in *B. distachyon* [13]. These results suggest that lysine succinylation plays both common and specific roles in different plant species.

## Conclusions

In this study, we identified 173 succinylated proteins with 330 unique modification sites in common wheat through MS analysis. The identified proteins are localized in multiple compartments and belong to diverse functional groups, suggesting that lysine succinylation plays important roles in regulating numerous biological processes. Overlap between lysine succinylation and acetylation indicates coordination between these two post-translational modifications. Comparative analysis showed that lysine succinylation is conserved between common wheat and *Brachypodium distachyon*. Our findings reinforce the notion that lysine succinylation plays critical regulatory roles in diverse aspects of cellular metabolism, especially in photosynthesis and Calvin-Benson cycle. The dataset may serve as a rich resource that can be used to examine the functions of lysine succinylation in this globally important cereal crop.

## Additional files

Additional file 1: Figure S1. Proteome-wide identification of lysine succinylation sites in common wheat. **Figure S2.**Succinylation of catalase 1. **Figure S3.** Secondary structure analysis of succinylated proteins. **Figure S4.** Domain-based enrichment analysis and KEGG pathway-based enrichment analysis of succinylated proteins. **Figure S5.**Interaction network of succinylated proteins associated with citrate, ribosome, oxidative phosphorylation and plant-pathogen interaction. **Figure S6.**Overlap between succinylation and acetylation in proteins involved in carbon fixation in common wheat. (DOC 7985 kb)

Additional file 2: Table S1. The identified lysine succinylation sites in common wheat. **Table S2.** Protein GO enrichment based on biological process. **Table S3** Protein GO enrichment based on molecular function. **Table S4.**. Protein GO enrichment based on cellular component. **Table S5.** Protein domain enrichment analysis. **Table S6.** Protein pathway enrichment analysis. **Table S7.** Protein interaction network of acetylated proteins. **Table S8.** Information of protein interaction networks. **Table S9.** 33 common elements in “acetylated protein” and “succinylated protein”. **Table S10.** 21 common sites in acetylation and succinylation. **Table S11.** Homologous succinylated proteins identified in common wheat and *Brachypodium distachyon*. **Table S12.** KEGG pathway analysis of the homologous succinylated proteins identified in common wheat and *Brachypodium distachyon*. (XLS 583 kb)

## Abbreviations

AGC: Automatic gain control
DTT: Dithiothreitol
FDR: False discovery rate
FNR: Ferredoxin-NADP^+^ oxidoreductase
GO: Gene ontology
HPLC: High performance liquid chromatography
KEGG: Kyoto encyclopedia of genes and genomes
LC-MS/MS: Liquid chromatography-mass spectrometry
PTMs: Post-translational modifications
