# Supplementary material for: Global analysis of protein lysine succinylation profiles in common wheat
Source: BMC Genomics. 2017 Apr 20;18:309. doi: 10.1186/s12864-017-3698-2 (PMC5397794; doi:10.1186/s12864-017-3698-2)
Supplement: Supplementary file 1 — Proteome-wide identification of lysine succinylation sites in common wheat. Figure S2.Succinylation of catalase 1. Figure S3. Secondary structure analysis of succinylated proteins. Figure S4. Domain-based enrichment analysis and KEGG pathway-based enrichment analysis of succinylated proteins. Figure S5.Interaction network of succinylated proteins associated with citrate, ribosome, oxidative phosphorylation and plant-pathogen interaction. Figure S6.Overlap between succinylation and acetylation in proteins involved in carbon fixation in common wheat. (DOC 7985 kb) [file 12864_2017_3698_MOESM1_ESM.doc]

**Global analysis of protein lysine succinylation profiles in common wheat**

Yumei Zhang^1 †^ , Guangyuan Wang^2 †^ , Limin Song^1^ , Ping Mu^1^, Shu Wang^3^ , Wenxing Liang^1 *^, Qi Lin^1*^

**Figure S1.** Proteome-wide identification of lysine succinylation sites in common wheat

**Figure S2.** Succinylation of catalase 1.

**Figure S3.** Secondary structure analysis of succinylated proteins

**Figure S4.** Domain-based enrichment analysis and KEGG pathway-based enrichment analysis of succinylated proteins

**Figure S5.** Interaction network of succinylated proteins associated with citrate, ribosome, oxidative phosphorylation and plant-pathogen interaction

**Figure S6.** Overlap between succinylation and acetylation in proteins involved in carbon fixation in common wheat

**Figure S1.** Proteome-wide identification of lysine succinylation sites in common wheat. **a** Overview of experimental procedures used in this study. **b** Mass error distribution of all identified peptides. **c** Peptide length distribution. **d** Succinyl-proteins based on the number of succinylated peptides that they contained

**
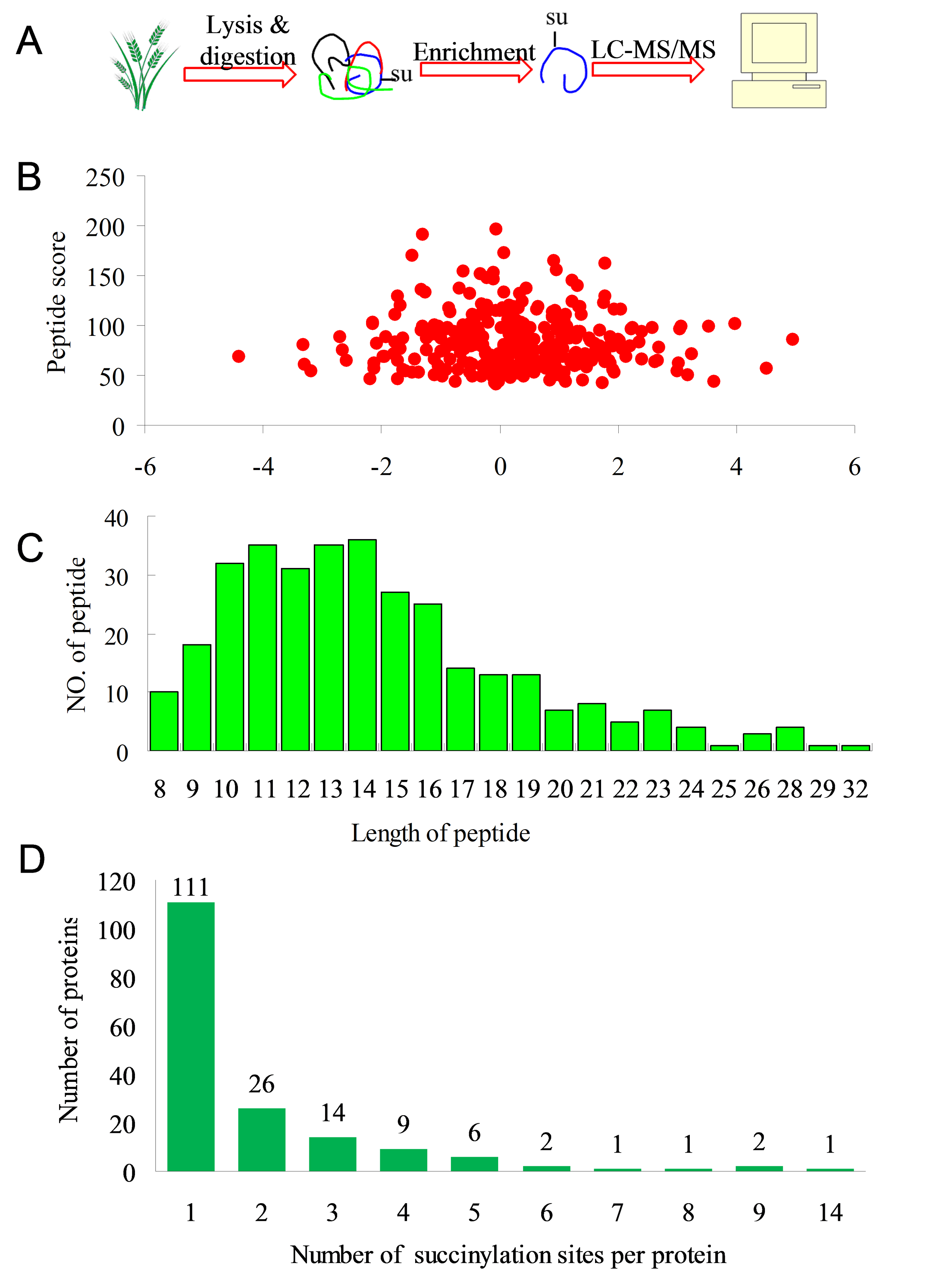
**

**Figure S2.** Succinylation of catalase 1. **a** MS/MS spectrum of succinyl-peptide SLGQK(su)LASR with a succinylation site at K481. **b** Validation of lysine succinylation of catalase 1 by Western blot analysis. Immunoprecipitation of catalase 1 was performed with (+) or without (-) catalase 1 antibody (Ab) and the eluted proteins were probed with either anti-succinyl lysine antibody (suK) or catalase 1 antibody

**Figure S3.** Secondary structure analysis of succinylated proteins. **a** Probabilities of lysine succinylation in the structures of alpha-helix, beta-strand and coil. **b** Predicted surface accessibility of succinylation sites

**
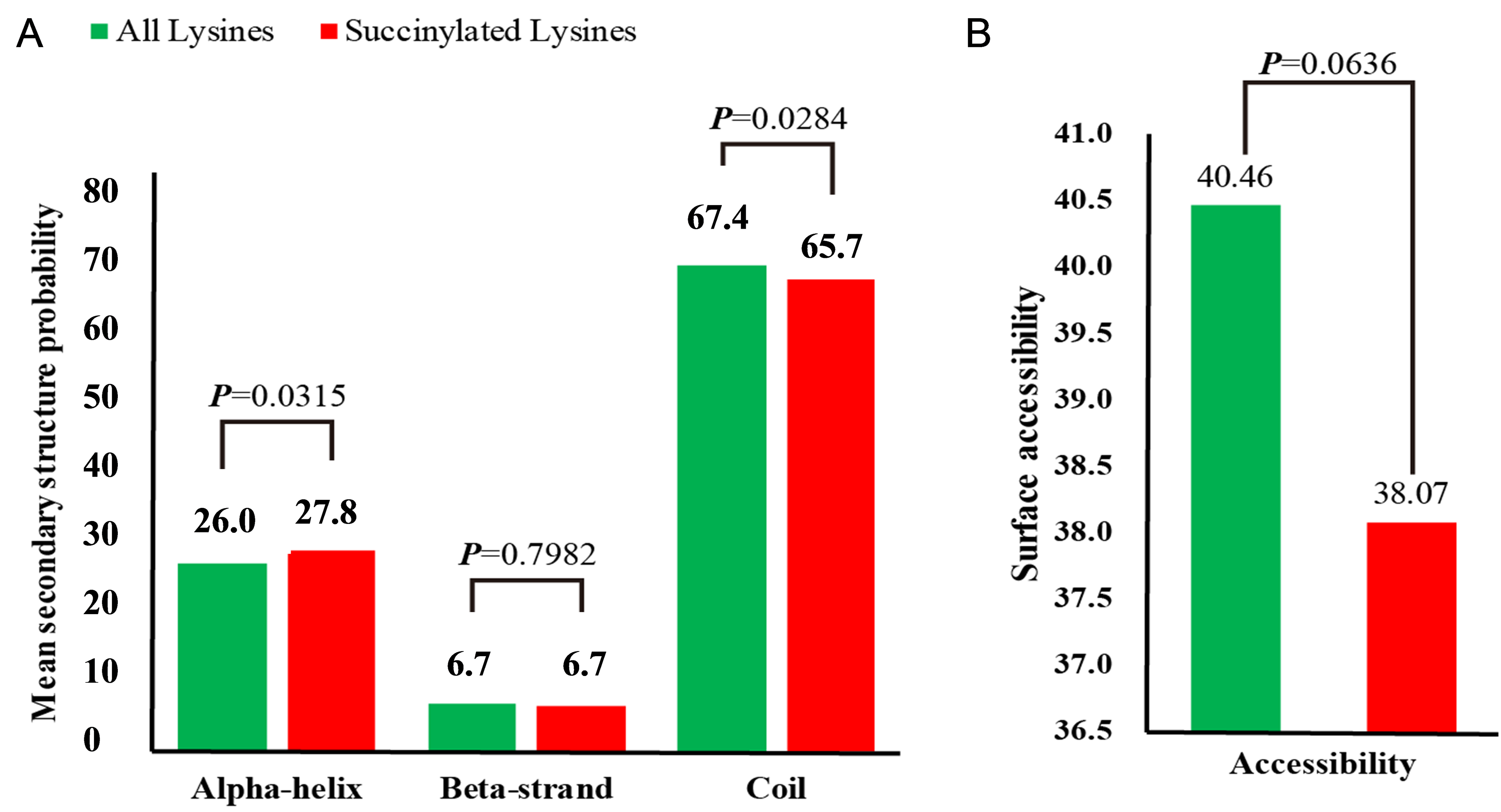
**

**Figure S4.** Domain-based enrichment analysis (**a**) and KEGG pathway-based enrichment analysis of succinylated proteins (**b**).


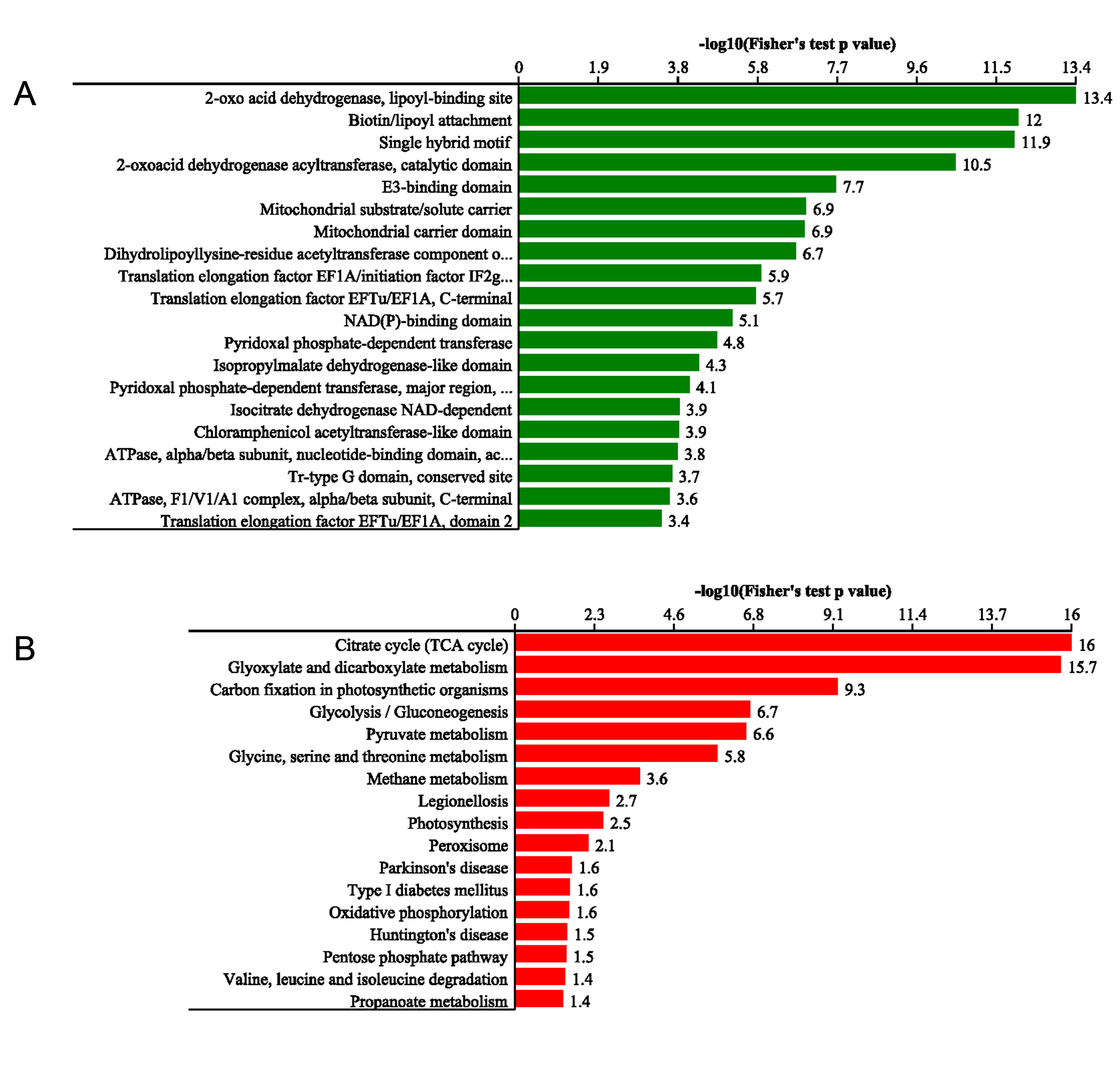


**Figure S5.** Interaction network of succinylated proteins associated with citrate (**a**), ribosome (**b**), oxidative phosphorylation (**c**) and plant-pathogen interaction (**d**).


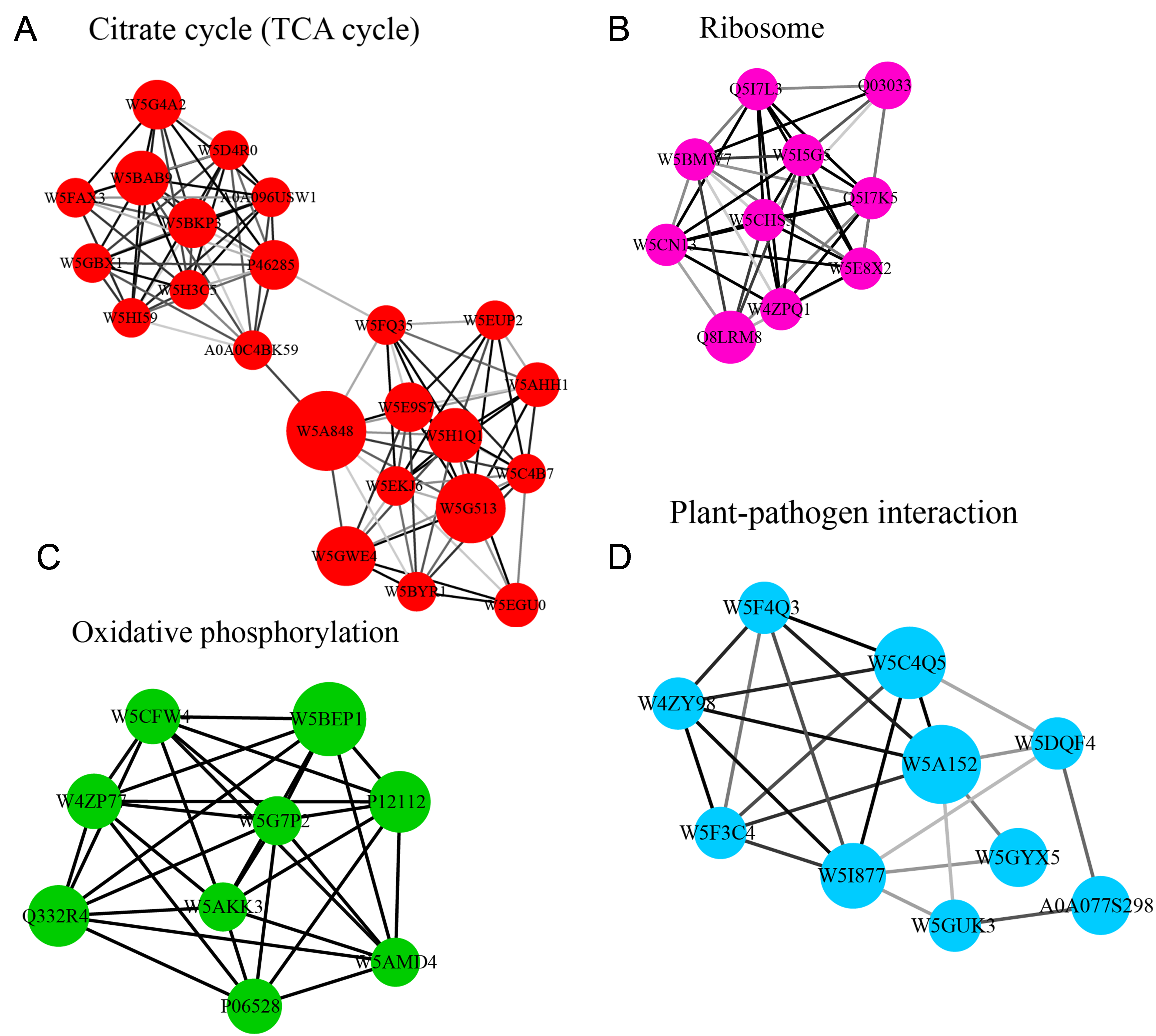


**Figure S6.** Overlap between succinylation and acetylation in proteins involved in carbon fixation in common wheat. Identified acetylated proteins were highlighted in yellow and identified succinylated proteins were marked by red-triangle.

**
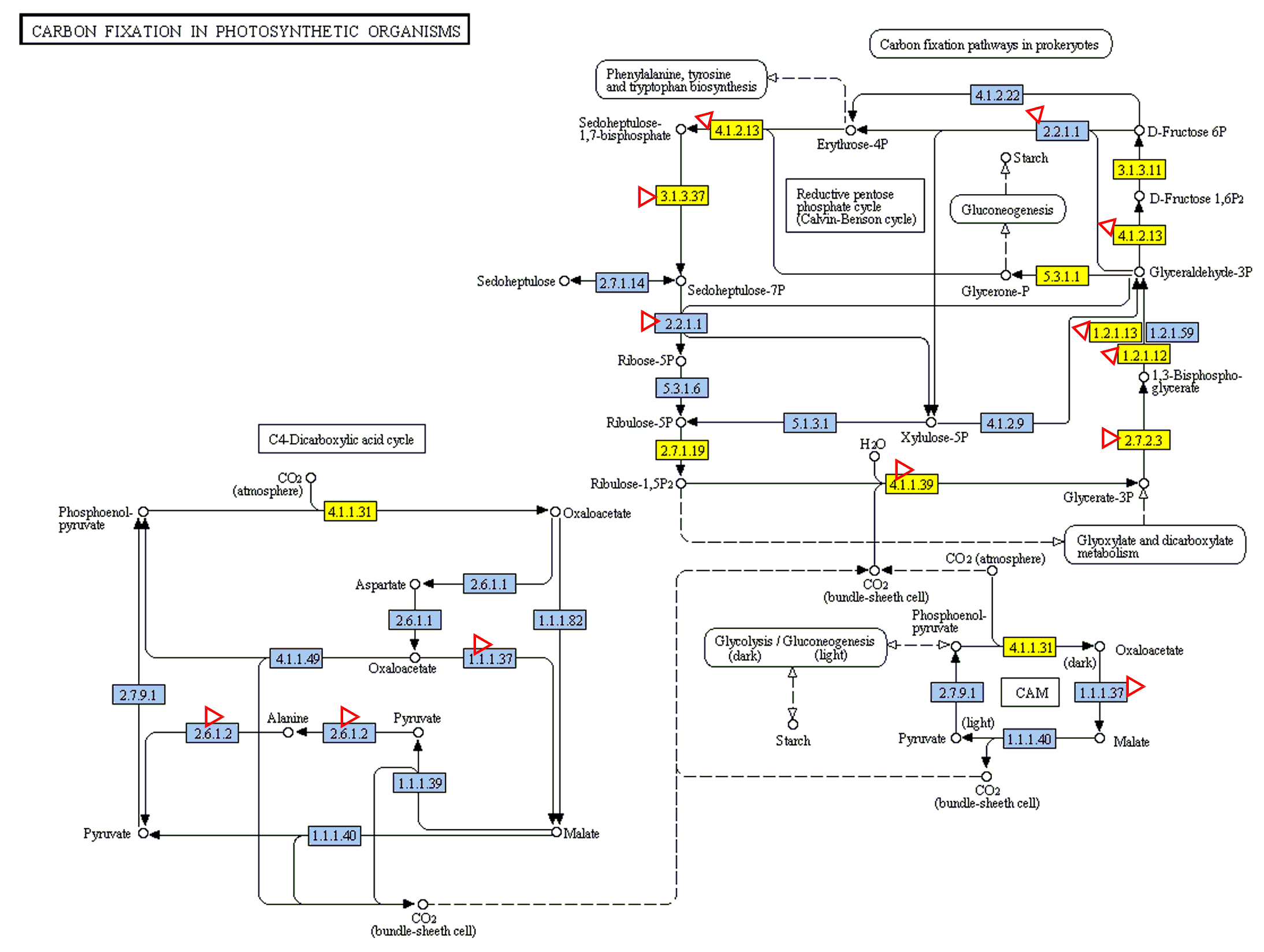
**
